# Non-contrast MRI of Inner Ear Detected Differences of Endolymphatic Drainage System Between Vestibular Migraine and Unilateral Ménière's Disease

**DOI:** 10.3389/fneur.2022.814518

**Published:** 2022-04-29

**Authors:** Yangming Leng, Ping Lei, Cen Chen, Yingzhao Liu, Kaijun Xia, Bo Liu

**Affiliations:** ^1^Department of Otorhinolaryngology, Union Hospital, Tongji Medical College, Huazhong University of Science and Technology, Wuhan, China; ^2^Department of Radiology, Union Hospital, Tongji Medical College, Huazhong University of Science and Technology, Wuhan, China

**Keywords:** vestibular migraine, Ménière's disease, magnetic resonance imaging, endolymphatic sac, endolymphatic duct

## Abstract

**Objective:**

We aimed to evaluate the diagnostic performance of some anatomical variables with regard to endolymphatic sac (ES) and duct (ED), measured by non-contrast three-dimensional sampling perfection with application-optimized contrasts using different flip angle evolutions (3D-SPACE) magnetic resonance imaging (MRI), in differentiating vestibular migraine (VM) from unilateral Ménière's disease (MD).

**Methods:**

In this study, 81 patients with VM, 97 patients with unilateral MD, and 50 control subjects were enrolled. The MRI-visualized parameters, such as the distance between the vertical part of the posterior semicircular canal and the posterior fossa (MRI-PP distance) and visibility of vestibular aqueduct (MRI-VA), were measured bilaterally. The diagnostic value of the MRI-PP distance and MRI-VA visibility for differentiating VM from unilateral MD was examined.

**Results:**

(1) Compared with the VM patients, patients with unilateral MD exhibited shorter MRI-PP distance and poorer MRI-VA visibility. No differences in the MRI-PP distance and MRI-VA visibility were detected between patients with VM and control subjects. (2) No significant interaural difference in the MRI-PP distance and MRI-VA visibility was observed in patients with VM and those with unilateral MD, respectively. (3) Area under the curve (AUC) showed a low diagnostic value for the MRI-PP distance and MRI-VA visibility, respectively, in differentiating between the VM and unilateral MD.

**Conclusions:**

Based on non-enhanced MRI-visualized measurement, anatomical variables with regard to the endolymphatic drainage system differed significantly between the patients with VM and those with unilateral MD. Further investigations are needed to improve the diagnostic value of these indices in differentiating VM from unilateral MD.

## Introduction

Two distinct clinical entities, vestibular migraine (VM) and Ménière's disease (MD), remain the frequent causes of the episodic vestibular syndrome (1, 2). VM is a relatively new disorder that is characterized by episodic vertigo or dizziness and coexisting migraine. MD is presented as the episodic vertigo attack, fluctuating sensorineural hearing loss (SNHL), tinnitus, and aural fullness, and the pathology of MD is characterized by endolymphatic hydrops (ELH). The linkage between migraine and MD was proposed as early as 1861 until the recent establishment of a definitive association (3). Considerable overlap of symptoms has been reported in MD and VM, such as vertigo, migraine, hearing loss, and tinnitus (2). Ghavami et al. found that, in patients with definite MD, 95% had one or more features of migraine, 51% had migraine headaches, and 48% met the diagnostic criteria of VM (4). Various objective approaches have been reported to identify VM and MD, such as caloric response, video head impulse test (5), vestibular evoked myogenic potentials (VEMPs) (6), biological markers (7), motion perception thresholds (8), and gadolinium-enhanced magnetic resonance imaging (MRI) of the inner ear (9). Even so, there are no pathognomonic findings for VM or MD, and no clinical test can fully differentiate between these two conditions. For instance, the shifts of VEMPs threshold and tuning can be found both in patients with MD and those with VM, which suggests that VM might share a common pathophysiology with MD (10–12). Additionally, about 8–25% of patients with VM have unilateral vestibular hyporeflexia to caloric irrigation (13–16), while the incidence of attenuated caloric response in patients with MD ranges somewhere between 45 and 75% (15, 17–20). Recently, by using the intratympanic (21) or the intravenous (22) route of contrast agent application, MRI of the inner ear has been used to visualize ELH *in vivo* for patients with MD. However, this ELH *in vivo* can also be observed in the patients with VM (23, 24). Therefore, the clinical discrimination between VM and MD remains challenging due to the overlapping clinical criteria and the lack of selective and sensitive diagnostic tools (25–27).

The pathophysiology of VM and MD has yet to be completely elucidated. The variability of symptoms and clinical findings both during and between attacks in patients with VM suggests that migraine affects the vestibular system at multiple levels. The presumed mechanisms comprised the cortical spreading depression, genetic defects, neurotransmitters modulation, the reciprocal connections between the trigeminal and vestibular nuclei, and etc. (28). Alternatively, the pathological hallmark of MD is ELH. At present, it is generally believed that ELH arises from the increased endolymph production or decreased endolymph absorption. Many factors have been proposed as leading to the development of ELH, which involve anatomical abnormalities, ionic imbalance, genetic predisposition, autoimmune reactions, viral infection, vascular irregularities, allergic responses, among others (29, 30). As for the anatomical variations of the inner ear, histopathological studies have revealed that patients with MD have significantly smaller vestibular aqueducts (VA) and endolymphatic sacs (ES) than healthy individuals (31, 32). Moreover, numerous radiological studies using MRI and computerized tomography (CT) have confirmed the presence of anatomical variations of inner ear in patients with MD (33, 34). These radiological variations include a significantly reduced distance between the vertical part of the posterior semicircular canal and the posterior fossa (33, 35), less visibility of VA and endolymphatic duct (ED) (34), poorer periaqueductal pneumatization (36), higher prevalence of jugular bulb abnormalities (37), retro-vestibular bony hypoplasia (38), and so on. The anatomical abnormalities of the inner ear do not seem to correspond to the existing pathophysiology of VM.

Previous studies have analyzed the differences between patients with VM and MD in terms of clinical presentation, audio-vestibular function, and inner ear MRI with gadolinium (5–7, 9). However, until now, to our knowledge, no study has examined the significance of the anatomical variations of inner ear associated with the ES and ED in differentiating between these two diseases. In this retrospective study, we looked into the radiological indices of inner ear based on the MRI-visualized measurement in patients with VM, unilateral MD, and control subjects. We sought to determine whether these radiological variations are helpful for differentiating VM from MD.

## Materials and Methods

### Participants

This retrospective study was conducted in the Union Hospital of Tongji Medical College, Huazhong University of Science and Technology, Wuhan, China.

In this study, eighty-one patients with VM and ninety-seven patients with unilateral definitive MD were enrolled between August 2016 and July 2020. Definite and probable VM was diagnosed against the International Headache Society (IHS) (25) and Bárány Society criteria (26), respectively. Furthermore, the diagnosis of unilateral definitive MD was in accordance with the diagnostic criteria proposed by the Bárány Society (39). For all patients, a thorough history investigation, otoscopy, neurotological examinations (audiometry, impedance, videonystagmography, caloric test, etc.), and imaging evaluations were conducted for differential diagnosis. In addition, fifty control subjects without audio-vestibular symptoms were enrolled.

Exclusion criteria were: (1) VM and MD co-morbidities; (2) middle or inner ear infections (otitis media, mastoiditis, labyrinthitis, etc.); (3) middle or inner ear anomaly (common cavity malformation, semicircular canal dysplasia, enlarged vestibular aqueduct, etc.); (4) bilateral MD; (5) having received previous otologic surgery or intratympanic injections; (6) retro-cochlear lesions (vestibular schwannoma, internal acoustic canal stenosis, etc.); and (7) head trauma.

This study was conducted according to the tenets of the Declaration of Helsinki. Informed consent was obtained from each patient and control. The project was approved by the ethical committee of the Tongji Medical College of Huazhong University of Science and Technology.

### Audio-Vestibular Evaluations

All patients received audio-vestibular evaluations during the interictal period, such as the pure tone audiogram and caloric test. Within 48 h before testing, all subjects were instructed to refrain from alcohol, caffeine, or medications (sedative, anti-depressant drugs, etc.) that would affect the results of vestibular tests.

### Radiological Evaluations

All participants received MRI examinations by the Verio or Magnetom Trio 3T scanners (Siemens, Erlangen, Germany) with a 12-element phased array coil. T1-weighted and T2-weighted imaging were applied. Three-dimensional sampling perfection with application optimized contrasts using different flip angle evolutions (3D-SPACE) was used to measure the distance between the vertical part of the posterior semicircular canal and the posterior fossa (Supplementary Table 1).

**Figure 1:**
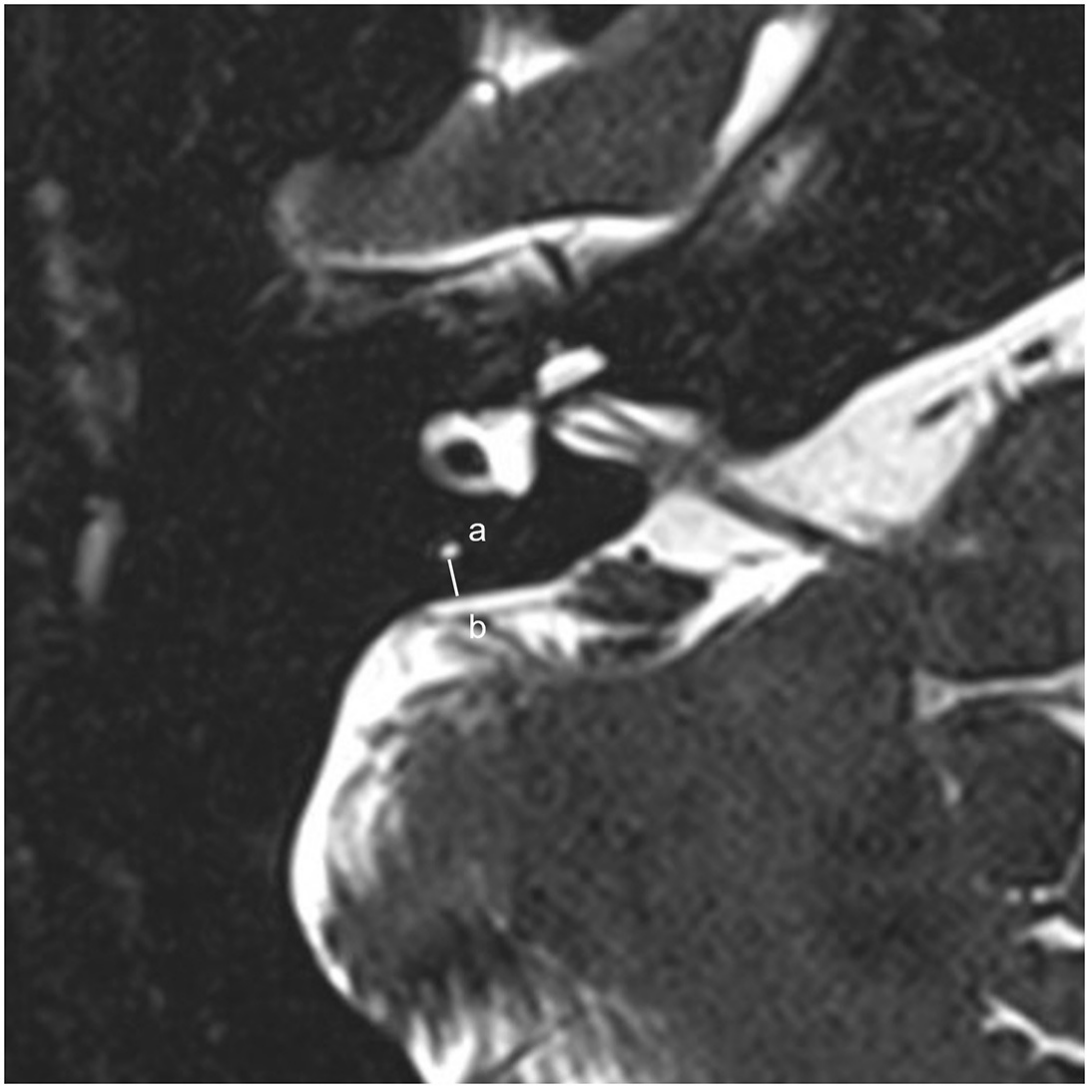
A 0.5-mm axial 3D-SPACE MRI scan showing detailed image of the right ear at the level of the measured distance between the vertical part of the posterior semicircular canal **(a)** and the posterior fossa **(b)**. *3D-SPACE*, three-dimensional sampling perfection with application optimized contrasts using different flip angle evolutions.

**Figure 2:**
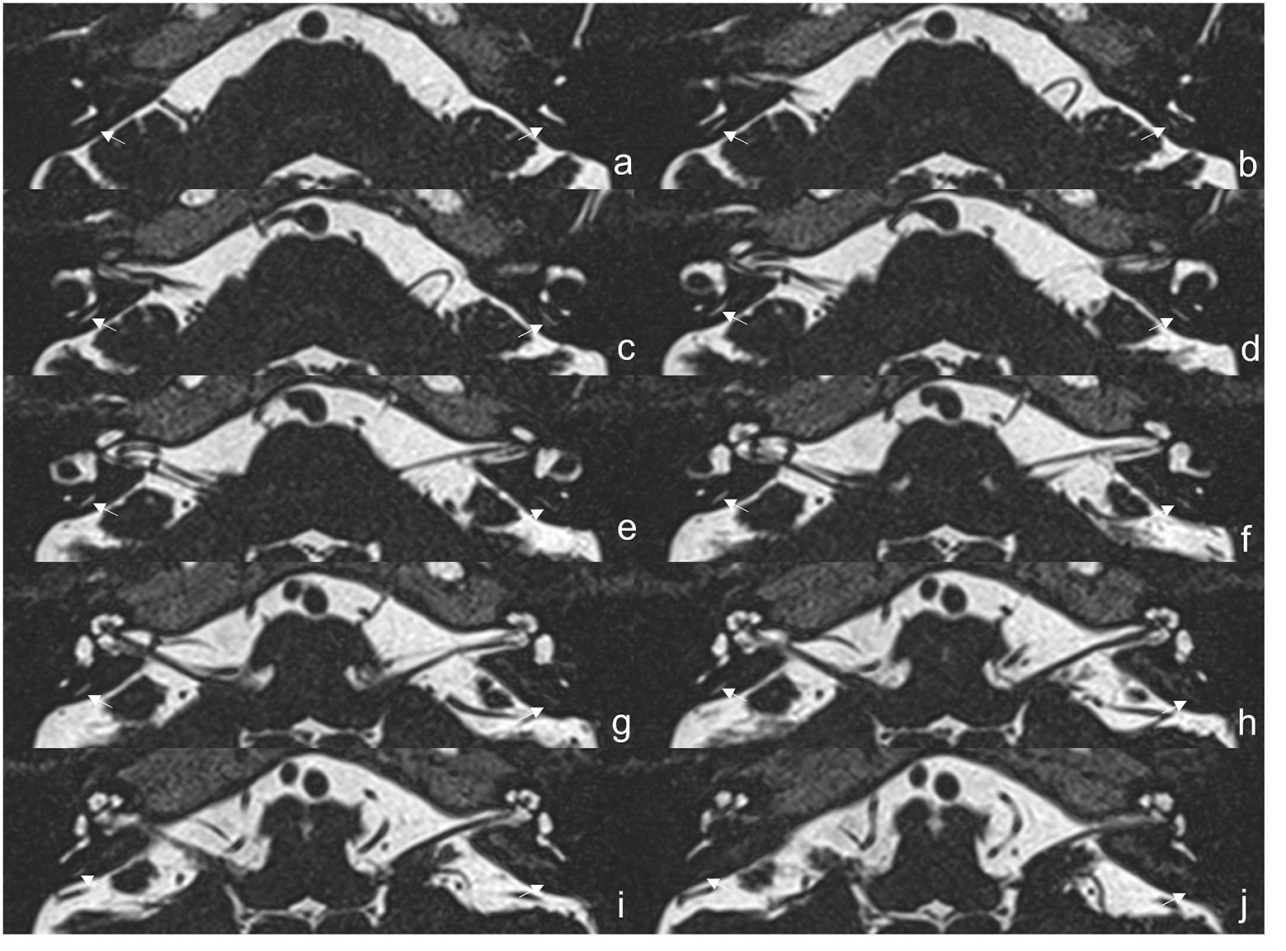
The 3D-SPACE MRI images of a 57-year-old female with vestibular migraine (VM). **(a–j)** Axial, high-resolution, and T2-weighted MRI scan showing visualization of the vestibular aqueduct on both sides. *3D-SPACE*, three-dimensional sampling perfection with application optimized contrasts using different flip angle evolutions, *VM*, vestibular migraine.

**Figure 3:**
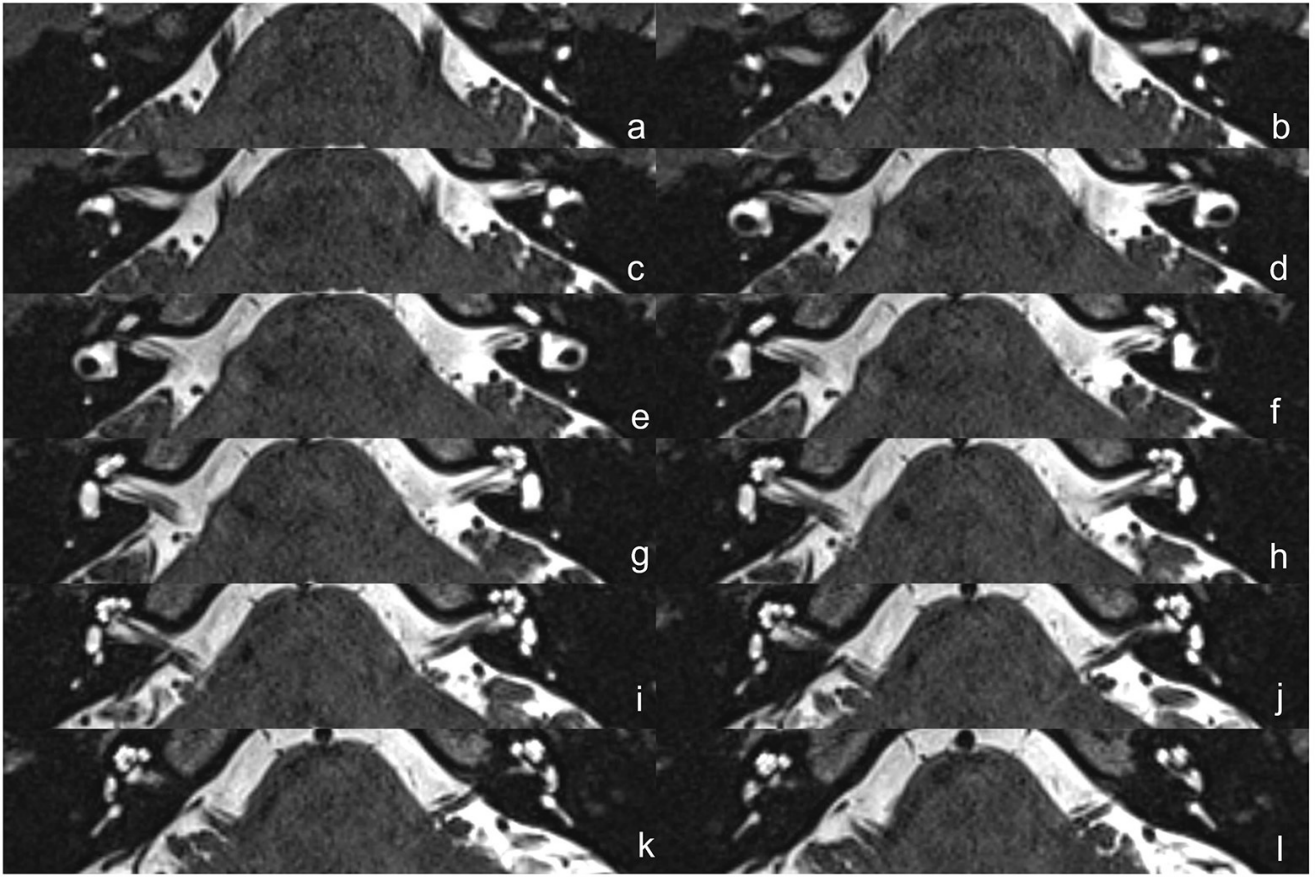
The 3D-SPACE MRI images of a 48-year-old female with VM. **(a–l)** Axial, high-resolution, and T2-weighted MRI scan showing non-visualization of the vestibular aqueduct on both sides. *3D-SPACE*, three-dimensional sampling perfection with application optimized contrasts using different flip angle evolutions, *VM*, vestibular migraine.

The protocol of radiological evaluations has been detailed in our most recent report (40). All radiological data were transferred to the workstations, and imaging analyses were performed on a Picture Archiving and Communication System (PACS) workstation (Carestream Client, Carestream Health, Rochester, NY, USA). Radiological data of all subjects were intermixed and reviewed by two senior neuroradiologists who were blinded to the clinical data (L.P with an experience of over 10 years and C.C over 5 years). In this study, the involved anatomical variables by MRI-visualized measurement included the distance between the vertical part of the posterior semicircular canal and the posterior fossa (MRI-PP distance, as presented in Figure 1) and visualization of VA (MRI-VA visibility). Visibility of VA refers to a linear or dot-like high intensity that is visualized continuously on more than one MRI sections in the direction of common crus to the posterior edge of the temporal bone. Figures 2, 3 presented the typical examples of visualization and non-visualization of VA in 3D-SPACE, respectively.

### Statistical Analysis

Statistical analyses were performed by using software SPSS (version 22.0). All continuous variables are presented as means ± standard deviations (SDs) or median and interquartile range (IQR 25–75th percentiles) after verification of normal distribution. Categorical variables are presented as counts and percentages. Data were tested for normal distribution using the Shapiro–Wilk test. The Mann–Whitney *U*-test was used for comparison between two groups and the Kruskal–Wallis *H*-test for more than two groups. A chi-square test was performed for categorical variables. The interobserver agreement for MRI-measurement was determined using the intraclass correlation coefficient (ICC). The agreement was generally interpreted as: poor, ICC <0.20; fair, 0.2 < ICC ≤ 0.40; moderate, 0.4 < ICC ≤ 0.60; good, 0.6 < ICC ≤ 0.80; and excellent, 0.8 < ICC ≤ 1.0. The significance level was set at 0.05.

The diagnostic value of the radiological data was characterized by using a receivers operating characteristic (ROC) curve. When a significant cutoff value was observed, the sensitivity, specificity of MRI-PP distance, and MRI-VA visibility for differentiating the affected sides of MD, VM, and those of control subjects were calculated. Area under the curve (AUC) and 95% confidence intervals (*CI*s) were estimated for the diagnostic value of radiological data. The meaning of AUC is defined as: no diagnostic value if AUC < 0.5, low diagnostic value if AUC is between 0.5 and 0.7, moderate diagnostic value if AUC is between 0.7 and 0.9, and high diagnostic value if AUC > 0.9.

## Results

### Demographic Characteristics of the Participants

In the VM group, 81 patients (57 cases of definitive VM and 24 cases of probable VM) were included, of which 70 (86.4%) were women and 11 (13.6%) were men. The average age was 42.93 ± 10.45 years old. Of these patients with VM, 48 cases (59.3%) that manifested episodic vertigo, 31 cases (38.3%) who had positional vertigo, and 37 cases (45.7%) had intolerance to head movement. In addition, 28 cases (34.6%) exhibited cochlear symptoms (subjective hearing loss, tinnitus, or aural fullness), 54 patients (66.7%) had photophobia, 52 patients (64.2%) showed phonophobia, and 51 case (63.0%) experienced motion sickness. All patients with VM underwent caloric test, and 20 cases (24.7%) showed an abnormal canal paresis (CP) value in one ear.

In the unilateral definitive MD group, 97 patients were involved, of which 53 (54.6%) were women and 44 (45.4%) were men. The average age was 48.20 ± 12.55 years old. Furthermore, 50 healthy subjects (40 women and 10 men) were enrolled as a control group, with an average age of 50.44 ± 12.59 years old.

In this study, the interobserver agreement for radiological assessment was excellent for MRI-PP distance (ICC = 0.981) and MRI-VA visibility (ICC = 0.846), respectively. Therefore, the results evaluated by one neuroradiologist were used randomly for further analyses.

### Radiological Variations in Patients With VM and Unilateral MD

**Figure 4:**
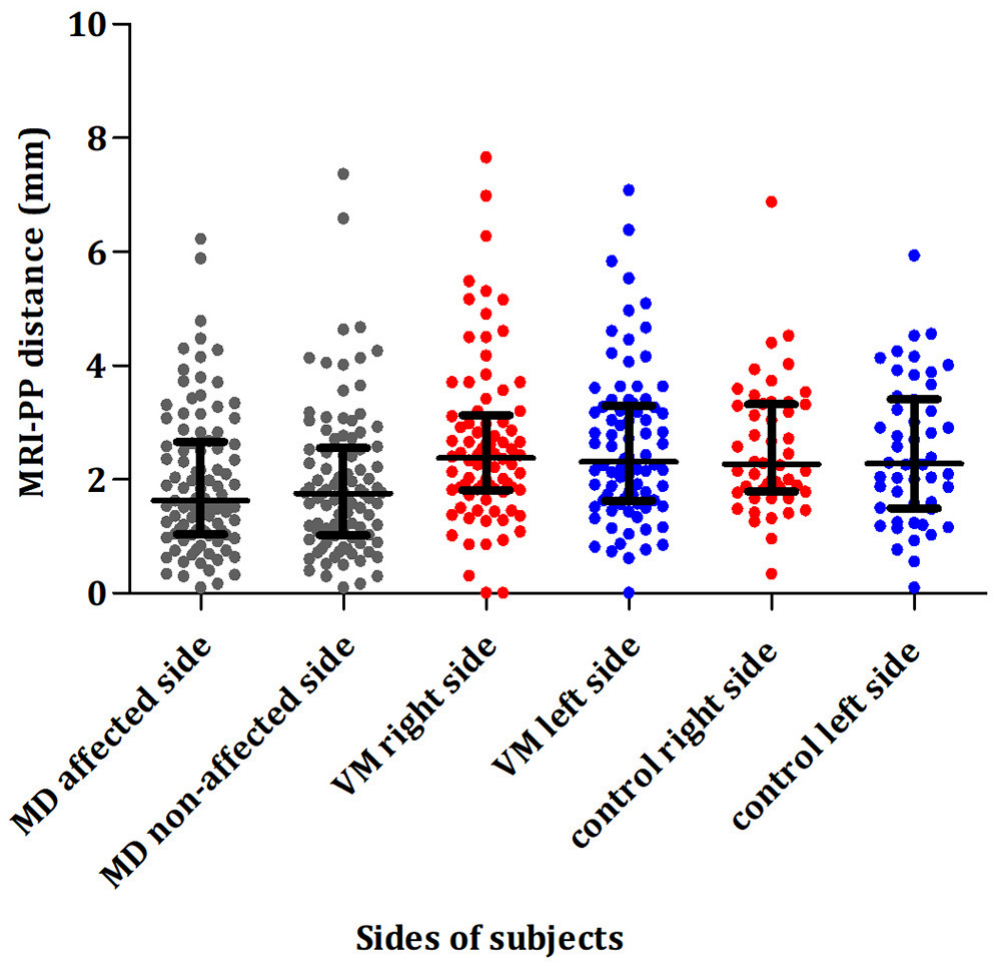
Comparison of the distance between the vertical part of the posterior semicircular canal and the posterior fossa (MRI-PP) distance in patients with unilateral MD (affected and non-affected side), patients with VM (right and left side), and control subjects (right and left side). *MD*, Ménière's disease; *VM*, vestibular migraine.

Of 81 patients with VM, the left and right ears had a median MRI-PP distance of 2.26 (1.61, 3.276) and 2.36 (1.805, 3.06) mm, respectively. The percentage of MRI-VA visibility in the left and right sides was 42% (34/81) and 37% (30/81), respectively. As shown in Figure 4, there was no significant interaural difference in MRI-PP distance or MRI-VA visibility in patients with VM (*Z* = 0.559, *p* = 0.576 and χ^2^ = 0.500, *p* = 0.481).

Of 97 patients with unilateral MD, the median MRI-PP distance in the affected and non-affected ears was 1.63 (1.04, 2.66) and 1.75 (1.02, 2.56) mm, respectively. The percentage of MRI-VA visibility in the affected and non-affected sides was 15.5% (15/97) and 19.6% (19/97), respectively. As shown in Figure 4, no significant differences in MRI-PP distance or MRI-VA visibility were found between the affected and non-affected side in patients with unilateral MD (*Z* = 0.103, *p* = 0.918 and χ^2^ = 0.643, *p* = 0.424).

Of 50 control subjects, the left and right ears had a median MRI-PP distance of 2.27 (1.79, 3.32) and 2.28 (1.50, 3.42) mm, respectively. The percentage of MRI-VA visibility in the left and right sides was 30% (15/50) and 28% (14/50), respectively. No significant interaural difference in MRI-PP distance or MRI-VA visibility was observed in control subjects (*Z* = 0.729, *p* = 0.466 and χ^2^ = 0.000, *p* = 1.000).

### Comparison of Anatomical Variations Among Three Groups

For comparison of the radiological indices, the left side of patients with VM and the left side of control subjects were randomly selected, along with the affected side of patients with MD. As for the MRI-PP distance, group comparison revealed significant difference among these three groups (χ^2^ = 13.250, *p* = 0.001). The results of pairwise comparisons between each two groups were as follows: (1) patients with MD showed shorter MRI-PP distance in the affected ears, compared with the left side of patients with VM (*p* = 0.002) and control subjects (*p* = 0.026), respectively. (2) No significant differences in MRI-PP distance were found between left side of VM and that of control subjects (*p* = 1.000). As for the MRI-VA visibility, group comparison revealed significant difference among these three groups (χ^2^ = 10.773, *p* = 0.005). Pairwise comparisons between each two groups were performed with a Bonferroni correction using an alpha level of 0.05/3 = 0.0167 and the results were as follows: (1) patients with MD showed poorer visibility of MRI-VA in the affected ears, compared with the left side of patients with VM (*p* = 0.001). (2) No significant differences in MRI-VA visibility were found between the left side of VM and that of control subjects (*p* = 0.293) and between the affected side of MD and left side of control subjects (*p* = 0.038).

### The Differential Diagnostic Value of Radiological Variations

**Figure 5:**
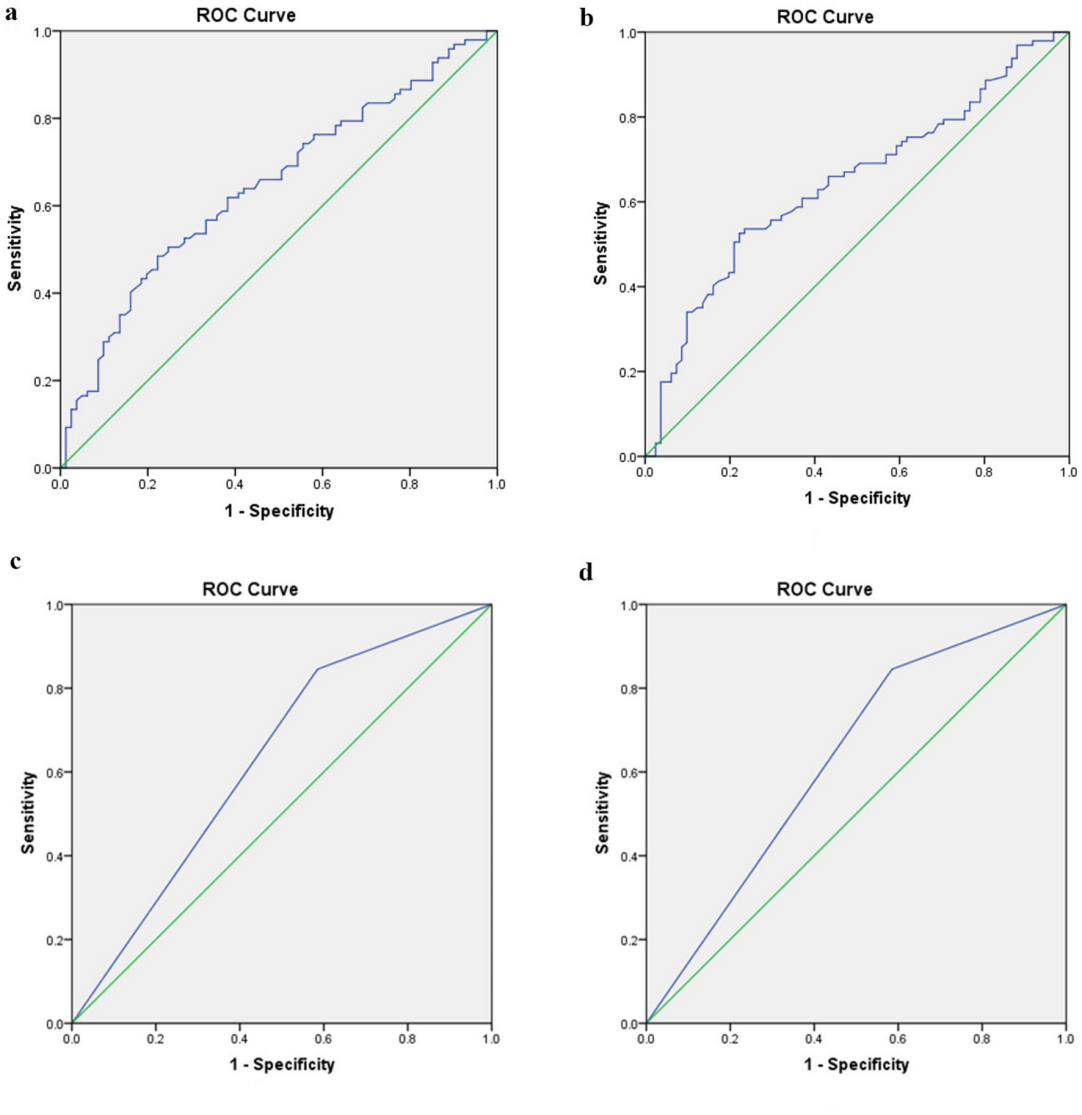
Receiver operating characteristic curves for the two radiological variables that showed significant difference between unilateral MD and VM. **(a)** Difference of MRI-PP distance between the affected side of MD and left side of VM. **(b)** Difference of MRI-PP distance between the affected side of MD and right side of VM. **(c)** Difference of MRI-VA visibility between the affected side of MD and left side of VM. **(d)** Difference of MRI-VA visibility between the affected side of MD and right side of VM. ROC, receivers operating characteristic; MD, Ménière's disease; VM, vestibular migraine; PP distance, distance between the vertical part of the posterior semicircular canal and the posterior fossa; VA, vestibular aqueduct.

When comparing the MD-affected side and the left side of VM, the AUC with 95% *CI* estimated for MRI-PP distance was 0.646 (0.566, 0.727). The ideal cutoff point was 1.56 mm, with sensitivity and specificity being 48.5 and 77.8%, respectively (Figure 5a). When comparing the MD-affected side and the right side of VM, the diagnostic value of the MRI-PP distance was also low with an AUC of 0.645 (0.564, 0.726). The cutoff MRI-PP distance of 1.7 mm had a sensitivity of 52.6% and specificity of 77.8% (Figure 5b).

When comparing the MD-affected side and the left side of VM, the AUC with 95% *CI* estimated for MRI-VA visibility was 0.630 (0.547, 0.713). The ideal cutoff point was 0.5, with sensitivity and specificity being 84.5 and 41.5%, respectively (Figure 5c). When comparing the MD-affected side and the right side of VM, the diagnostic value of the MRI-VA visibility was also low with an AUC of 0.589 (0.472, 0.707). The cutoff MRI-VA visibility of 0.5 had a sensitivity of 84.5% and specificity of 33.3% (Figure 5d).

## Discussion

### Differences of Radiological Variations of Inner Ear Between VM and MD

The present study showed that, compared with VM patients and control subjects, patients with unilateral MD had shorter MRI-PP distance and poorer MRI-VA visibility in both affected and non-affected ears. Meanwhile, no difference was found in MRI-PP distance and MRI-VA visibility between patients with VM and control subjects.

Anatomical variations of inner ear have been shown to play a role in the pathogenesis of MD (33, 34, 36–38), and morphological analysis by histopathological and radiological studies has confirmed hypoplasia of ED and ES as one of the predisposing factors. Radiologically, a short PP distance may suggest a small ES and poor ES function in patients with MD (33). In this study, another radiological variable was the visibility of VA in MRI. Previous histological studies have demonstrated hypoplasia of the VA and narrowing of the lumen of the ED in patients with MD, which may implicate congenital or developmental abnormality of the VA/ED as a likely predisposing factor for the development of ELH in patients with MD (32, 41, 42). Similar findings have been highlighted by 2D CT, 3D-Cone beam CT as well as by MRI (43–45). Another hypothesis to explain the calcification and narrowing of VA is calcium ion (Ca^2+^) augmentation in hydropic ears, as demonstrated in biological samples (46) and more recently with mineralized cells around the VA in histopathological analysis (47). ES and ED, as part of the endolymphatic drainage system, may play an essential role in maintaining endolymph homeostasis. Pathophysiologically, the hypoplasia of ES and ED has been assumed to compromise endolymph absorption, which could induce ELH in MD. To our knowledge, our study was the first to find a difference of MRI-visualized measurement in the endolymphatic drainage system between these two episodic vestibular syndromes, which indicated that the diminished endolymph absorption resulted from hypoplasia of the endolymphatic drainage system can be regarded as a predisposing factor in the pathogenesis of MD rather than VM.

As for the pathophysiology of VM, recent electrophysiological findings of caloric reflex and VEMP showed dysfunction of inner ear (10, 13, 48), which may be, at least partly, attributed to the neurogenic inflammation in the inner ear. Trigeminal nerve endings have been found in the blood vessels of the inner ear (49). Migraine attack or serotonin provocation could induce plasma extravasation from dural and labyrinth vessels, causing transient inflammation not only in the dura mater but also in the inner ear (50). Additionally, the migraine-associated nociceptive receptor has been observed in the human ES (51) and the absorption of the endolymph in the ES might be compromised in migraineurs. Furthermore, MRI-demonstratable ELH has been observed in the cochlear and/or vestibule of patients with VM (24, 52). These observations led to the hypothesis that MD and VM may share a common pathophysiology, i.e., ELH (10). From a radiological and anatomical perspective, our results supported these earlier findings by implying that different pathophysiological mechanisms are involved in the common condition ELH. Some factors other than a compromised endolymphatic drainage system may contribute to the pathogenesis of VM, as VM may affect the vestibular system at multiple levels, especially the central pathways (53). Based on these imaging discrepancies in the peripheral rather than the central vestibular system, our findings suggest that anatomical variations in the inner ear may play differential roles in the pathogenesis of VM and MD. These results might be used to develop more pathologically oriented diagnostic algorithms and strategies for treating these two conditions in the future.

### Differential Diagnostic Value of Radiological Variations Between VM and MD

The current study, from the ROC analyses, showed that the MRI-PP distance has a low diagnostic accuracy for discriminating unilateral MD from VM or controls, which means this radiological variation is not yet a suitable tool in the differential diagnosis between these two episodic vestibular syndromes.

Many studies have attempted to establish a method to distinguish MD from VM, which includes the history investigation, audio-vestibular testing, and imaging evaluation (5, 6, 9, 10, 54). But so far, no definite diagnostic test can reliably distinguish between these two entities. During the past two decades, high-resolution MRI with intravenous or intratympanic application of gadolinium as the contrast agent has provided direct evidence of ELH in the inner ear *in vivo* (21, 22), which was also used to differentiate VM from MD (9, 24, 52). Nakada et al. demonstrated that ELH *in vivo* was present in the vestibule in two out of seven patients with VM. Meanwhile, a significant unilateral or bilateral ELH can be found in the vestibule of all patients with MD (52). In addition, Sun et al. reported that the MRI-demonstratable ELH were observed in the cochlea and vestibule in the affected ears of patients with MD, while only suspicious cochlear hydrops and no vestibular hydrops was noted in the patients with VM (9). Nevertheless, Gürkov et al. found that 21% (4/19) patients with VM exhibited evidence of cochlear and vestibular ELH by enhanced MRI of the inner ear (24). The presence of MRI-demonstratable ELH *in vivo* in a small proportion of patients with VM could be attributed to neurogenic inflammation in VM, which could induce inner ear dysfunction and ELH. Another explanation might be the comorbidity of VM and MD.

Recent studies using other imaging modalities, such as position-emission tomography (PET) (55), blood-oxygen-level dependent functional MRI (BOLD-fMRI) (56), and MRI-based voxel-based morphometry (57), found that the enhanced vestibular organ perception and its interactions with the brainstem, thalamus, and cortex may underlie the pathogenesis of VM (58, 59). Furthermore, radiomics of the inner ear has been suggested as a promising tool in the diagnosis of MD (60). These findings, together with our results, implicated that although non-contrast MRI-based evaluations of the inner ear provides limited information in discriminating VM from MD, the radiological evidence of inner ear variations offers deeper insight into the pathophysiological differences of these two episodic vestibular syndromes. Future radiomic studies are expected to provide additional imaging evidence for the differential diagnosis of these two entities.

Our study has several limitations. First, this is a retrospective study and is potentially subjected to selection bias and information bias. Second, we did not analyze the radiological indices based on phenotypes of unilateral MD. The following five distinctive clinical subtypes have been identified by the Ménière's Disease Consortium (61): Type 1 included patients without a familial history of MD, migraine, or autoimmune comorbidity; Type 2 had delayed MD characterized by SNHL which antedated the vertigo episodes; Type 3 included all familial cases of MD; Type 4 was associated with migraine with or without aura, and Type 5 was defined by a concurrent autoimmune disorder (61). Recently, Diao et al. found that some radiological variables differed between patients with MD with and without migraine, including the poorer mastoid pneumatization and the shorter distance between the sigmoid sinus and posterior wall of the external acoustic canal (62). Then, it is reasonable to suppose that the anatomical variations may play inconsistent roles in different subtypes of unilateral MD. Large-scale study including full spectrum of MD subtypes are warranted in the future.

## Conclusions

Compared with VM patients, patients with definitive unilateral MD had a shorter MRI-PP distance and poorer visibility of MRI-VA in both affected and non-affected ears. The differences in these radiological indices between VM and MD may reflect different mechanisms underlying these two disease entities. However, these indices only showed low diagnostic value in differentiating VM from MD, which needs to be improved by further investigations.

## Data Availability Statement

The original contributions presented in the study are included in the article/Supplementary Material, further inquiries can be directed to the corresponding authors.

## Ethics Statement

The studies involving human participants were reviewed and approved by the Ethical Committee of Tongji Medical College of Huazhong University of Science and Technology. The patients/participants provided their written informed consent to participate in this study.

## Author Contributions

YLe: patient consultation, interpretation of data, drafting, and critical revision of the manuscript. PL and CC: data collection, image extraction, and analysis. YLi and KX: patient recruitment, data collection, and statistical analysis. BL: study conception and design, patient consultation, interpretation of data, and critical revision of the manuscript. All authors contributed to the article and approved the submitted version.

## Funding

This work was supported by grants from the National Natural Science Foundation of China (NSFC No. 81670930), the Natural Science Foundation of Hubei Province, China (No. 2016CFB645), and the Fundamental Research Funds for the Central Universities, China (No. 2016YXMS240).

## Conflict of Interest

The authors declare that the research was conducted in the absence of any commercial or financial relationships that could be construed as a potential conflict of interest.

## Publisher's Note

All claims expressed in this article are solely those of the authors and do not necessarily represent those of their affiliated organizations, or those of the publisher, the editors and the reviewers. Any product that may be evaluated in this article, or claim that may be made by its manufacturer, is not guaranteed or endorsed by the publisher.
