# Supplementary material for: Non-contrast MRI of Inner Ear Detected Differences of Endolymphatic Drainage System Between Vestibular Migraine and Unilateral Ménière's Disease
Source: Front Neurol. 2022 Apr 29;13:814518. doi: 10.3389/fneur.2022.814518 (PMC9099065; doi:10.3389/fneur.2022.814518)
Supplement: Supplementary file 1 [file Table_1.DOCX]

| **Supplementary Table 1**: MR scanning protocol | | | |
| --- | --- | --- | --- |
| Scanning Parameters（3.0T） | T1 weighted imaging | T2 weighted imaging | 3D-SPACE |
| Plane | sagittal and axial | axial | axial |
| TR (ms) | 300 | 9000 | 1000 |
| TE (ms) | 2 | 90 | 135 |
| Fat saturation | / | TIR | / |
| slice thickness(mm) | 5 | 5 | 0.5 |
| Slices(no.) | 15 | 17 | 56 |
| FOV (mm^2^) | 250×250 | 220×200 | 200×200 |
| matrix | 320×320 | 640×640 | 384×384 |
| Averages | 1 | 1 | 2 |
| Bandwidth (Hz/Px) | 330 | 287 | 289 |
| 3D-SPACE: Three-dimensional sampling perfection with application optimized contrasts using different flip angle evolutions; TR: Repetition Time; TE: Echo Time; FOV: Field of View. | | | |
|  |  |  |  |
|  |  |  |  |
